# Predicting Malaria Incidence in Northern and Northwestern, Pakistan

**Published:** 2018-12

**Authors:** Muhammad IMRAN KHAN, Humera QURESHI, Henock AMBACHEW, Hai-Feng PAN, Dong-Qing YE

**Affiliations:** 1. Dept. of Epidemiology and Biostatistics, School of Public Health, Anhui Medical University, 81 Meishan Road, Hefei, Anhui, China; 2. Anhui Province Key Laboratory of Major Autoimmune Diseases, 81 Meishan Road, Hefei, Anhui, China; 3. Dept. of Clinical Laboratory Diagnostics, First Affiliated Hospital, Anhui Medical University, Hefei, Anhui, China; 4. Dept. of Medical Laboratory Sciences, College of Medicine and Health Sciences, Hawassa University, Hawassa, Ethiopia

## Dear Editor-in-Chief

Malaria is one of the major public health problems which causes high mortality and morbidity in Pakistan, with an estimated 1.6 million cases reported annually (1). Malaria infections are predominantly falling in all age groups, but most are below five-year children, and pregnant women are vulnerable (2). Approximately 50 million women become pregnant every year in malaria high endemic areas. Resultantly, 10000 women and 200000 neonates deaths occurring from malaria complications during pregnancy (3). *Plasmodium vivax* and *Plasmodium falciparum* infections are prevalent in endemic areas of Pakistan (4). In Pakistan, several factors contribute and promote suitable environment for malaria transmission including agricultural practices with a vast irrigation network and monsoon rains; presence of population movements inside in the country and across international borders with Iran and Afghanistan (5); and weak socioeconomic condition in addition to natural disasters (2).

Month-wise malaria incidence obtained from the Directorate of Malaria Control, Pakistan from 2011 to 2015 and analyzed through Eviews-8 statistical software. The data consist of malaria positive cases reported in four endemic districts of northern (Lakki Marwat, Bannu), and northwestern (Kurram, Bajaur) Pakistan. These endemic districts are known as the most susceptible districts to malaria for almost hundred years back. Three time-series models carried out with stationary series of malaria cases for each district. Augmented Dickey-Fuller test used to check the time series data whether a series is stationary or not before using it to develop time series models. We identified autoregressive and moving average terms required by calculating the autocorrelation function (ACF) and partial autocorrelation function (PACF) through Correlogram for each model (6). After analyzing the models, the final and most crucial step is to check the model’s accuracy and compare them to select the best forecast model. In this regard, we use some useful measures to verify the model accuracy; highest stationary of R-Square, lowest value of AIC, and lowest value of MAE.

Our study revealed that transformation model had lowest AIC, MAE, and highest stationary R-square for all endemic districts. The terms autoregressive (AR) and moving average (MA) were significant (*P*<0.001) for all coefficients of transformation models. Hence, transformation model provides the accurate forecast for malaria incidence in endemic districts of northern and northwestern, Pakistan. In future, other studies should explore the above time series models with some metrological factors to improve the forecast accuracy for malaria incidence.
